# The Congress of American Physicians and Surgeons

**Published:** 1888-11

**Authors:** 


### THE CONGRESS OF AMERICAN PHYSICIANS AND SURGEONS.

---

*To the Editors of the Atlanta Medical and Surgical Journal:*

The Congress of American Physicians and Surgeons was a grand success, both socially and intellectually. Some of the leading medical and surgical lights of not only this, but the old country, were in attendance, among whom may be mentioned Prof. Von Esmarch, of Germany; Prof. Thomas Annandale, of Edinburgh; Sir Spencer Wells, Sir William MacCormac, Mr. Victor Horsley, Dr. William Ord, Dr. David Ferrier, Dr. Arthur Edward Durham and Dr. Wm. O. Priestly, the latter seven of London, England.

The general meeting to receive the report of the Executive Committee and for organization was held at 1:30 p. m., September 18th, in the Grand Army building, and was called to order by Dr. William Pepper, of Philadelphia, Chairman of the Executive Committee, who made a brief address, at the close of which he presented the President of the Congress, Dr. John S. Billings.

Dr. Samuel C. Busey, of Washington, delivered the address of welcome, in which he showered a wealth of words upon the beauties, attractions and advantages of Washington.

At the evening session of the Congress, some thoughtful, elaborate and highly interesting papers were read.

Dr. Reginald H. Fitz, of Boston, opened the discussion at the first evening session upon the Medical Relations of Acute Intestinal Obstruction. He said the object of his paper was to deal with the diagnosis and medical treatment of the acute internal, mechanical varieties of intestinal obstruction. The only causes recognized were strangulation from adhesions, vitelline remains, peritoneal slits, pockets and rings, intussusception, twists and knots, abnormal contents, strictures and tumors. The evidence presented resulted from an analysis of two hundred and ninety-five cases collected from medical literature since 1880.

The most constant symptoms of obstruction were shown to be pain, vomiting, tympany and tumor. Stoppage of the bowel was not regarded as the most essential symptom in diagnosis, since frequent loose stools characterize intussusception, and may occur in other varieties of obstruction.

The treatment consisted in the attempt to relieve intussusception of the large intestine by forced injection under anæsthesia, with massage, and to treat obstructing gall-stones by opium, possibly with the aid of laxatives and electricity. All the other varieties of acute obstruction require surgical treatment.

Dr. Nicholas Senn, of Milwaukee, contributed a paper on The Surgical Treatment of Intestinal Obstruction. He held that all cases of *true* intestinal obstruction are surgical affections. Intestinal obstruction is present when, from mechanical or dynamic causes, the intestinal canal becomes completely or partially imperforate, interfering completely or partially with fæcal circulation. It is important that a distinction be made between the cases due to mechanical causes and those due to dynamic causes. In the former the surgeon is expected to modify the mechanical cause; in the other the surgical treatment is limited to diminishing intra-abdominal pressure. *Manual exploration* by the rectum is a useful diagnostic and therapeutic measure where the obstruction is below the sigmoid flexure, provided the surgeon has a small hand. *Taxis and massage* should be limited to cases where the obstruction is due to intra-mural causes and to a few cases of invagination. Uniform, uninterrupted, equable pressure of the abdomen is useful in preventing hyperdistension in these cases, especially when dynamic causes are feared. *Enterotomy*, so frequently practiced in the past, he hoped had become very nearly obsolete. It should only be employed when it is clear that the patient will not be able to pass through the ordeal of laparotomy. *Lumbar colotomy.*—In cases where this operation was formerly employed we can often, by uniting the portion of the bowel above the obstruction with that below, by means of decalcified bone plates, avoid the necessity for colotomy. Dr. Senn exhibited specimens showing that a dilated congested bowel can be repaired when excluded from the fæcal circulation in this way. He had in the past eight months performed gastro-enterotomy in four cases by this method. The operation was a success in all the cases, although in one case the patient died because the operation had been postponed too long.

Dr. Arthur Edward Durham, of London, said there was no class of cases which are more serious or more dangerous than those of intestinal obstruction. The sooner the surgeon is called in these cases the better. As soon as the diagnosis is made the operation should be performed. In some cases the operation should be done before a *positive* diagnosis is reached in order to clear up the diagnosis, and under such circumstances the surgeon must be prepared to meet the indications that present themselves. In trying to reach a diagnosis the probabilities in favor of this or that condition, based upon statistics, should not influence us, but the case should be studied on its own merits and treated according to the indications presented. He would ask Dr. Senn whether it was not possible to blow up the patient by means of hydrogen gas, and would therefore suggest, as a substitute, atmospheric air.

Dr. William Ord, of London, admitted the great difficulty attending the diagnosis. In some cases the use of opium, or other narcotic, had relieved the obstruction. When, however, there was mechanical obstruction the case must sooner or later pass into the hands of the surgeon. The operation is a serious one, especially in people over fifty years of age.

Professor Thomas Annandale, of Edinburg, would divide cases of obstruction into two classes, the acute and the chronic. In acute cases medical measures should be tried, but not continued too long. If the symptoms are urgent, the surgeon should be called at the end of forty-eight hours, and the sooner the abdomen is opened the better. In chronic cases we may wait a considerable time, even until the symptoms become acute; the treatment should then be that applicable to a case of acute obstruction.

Dr. Senn assured Dr. Durham that there was not the least danger in the use of hydrogen gas. He had performed numerous experiments on animals and had demonstrated the procedure in man to be safe. In cases of intestinal perforation it aids the diagnosis, and allows the surgeon to determine whether or not all the openings have been closed. In a recent case of gunshot wound eleven openings near the ileocæcal valves were closed, and a twelfth was revealed by the hydrogen gas at the point where the peritoneum is reflected from the bowel to the bladder. The patient is doing well.

Dr. Roswell Park, of Buffalo, contributed a paper on Cerebral Localization in its Surgical Relations. With regard to where one could trephine with safety, the safest rule is to first apply the trephine over those areas which do not overlie large vascular channels. Afterwards the opening may be extended in any direction and to any required extent. The greatest hesitation is with regard to opening one of the sinuses. Two dangers attend such an accident—one fatal air embolism, the other profuse hemorrhage. The former danger is almost a theoretical one, and the other may be overcome by plugging the sinus or closing its wound with a fine needle and suture. The author had collected reports of 63 cases, 17 of which terminated fatally; 15 of the cases were abscesses, subdural or subcortical. In 11 cases the lesion was a tumor, exclusive of tubercular nodules. Of cysts there were 12 cases; the 25 other cases were of a mixed nature. Of the 63 operations, 17 were performed by American surgeons. MacEwan has operated 12 times; Horsley, 11; Bergmann, 4; Weir, 3; Keen, 3, and Park, 3.

Dr. Charles K. Mills, of Philadelphia, read an elaborate paper on Cerebral Localization in its Practical Relations, in which he alluded to the discoveries of Bouillaud and Broca on speech localization; to the announcement by J. Hughlings Jackson in 1864, that certain convolutions superintended the delicate movements of the hand, which were under the immediate control of the mind. In considering the localization of brain-tumors, he referred to twenty cases of autopsies occurring in his own personal experience, in about one-half of which the tumors were in surgically accessible areas, and in at least one-fourth of which successful operations might have been performed. True Jacksonian epilepsy was sometimes reflex in origin—that is, it is established by intense, persistent, peripheral irritation, and even after the irritation had been removed the cortical discharge continued; herein, perhaps, lay the explanation of Jacksonian spasm without coarse lesion herein; also, perhaps, was to be found the justification for the excision of the cortical discharging areas.

Dr. David Ferrier had long cherished the idea that the determination of the functions of the brain would in time lead to the successful treatment by surgery of some of the most distressing ailments of our fellow-creatures. When he first broached the possibility of this he received little encouragement, but now cerebral surgery had become a distinct branch of the art of surgery.

Mr. Victor Horsley described the results of his experiments at length upon the motor region. Here, he believed, three functions were clearly represented: *First*, the representation of so-called tactile sense; *second*, representation of the so-called motor sense; and *third*, the great representation of movement. It is found that, morphologically, the large cells in the fourth layer are concerned in the representation of movement, and he could not understand that we should not allot to the small cells in the upper layer the representation of sensation. Experimentation shows that the convulsions of so-called Jacksonian epilepsy are solely due to the cortex, and not at all dependent upon the spinal cord or upon the bulbar spinal system.

Dr. W. W. Keen, of Philadelphia, compared the head cavity to the other cavities of the body. The brain may be considered to be made up of a number of viscera, having separate and distinct functions, and each of which has its own physical signs and symptoms. The timidity with which the surgeon formerly approached the abdominal cavity was remarkable; the boldness with which we now attack this cavity is almost appalling and the success equally gratifying. This history will be repeated in the case of the brain.

Dr. Robert F. Weir, of New York, since 1883, had operated in ten cases of brain surgery—three times for tumor, three for abscess, twice for hemorrhage into the cerebrum where there was no external injury to indicate its locality, once for epilepsy and once for cerebral pain. Keen has removed a tumor weighing over four ounces from the brain; Horsley has removed with success one weighing four and a half ounces from a patient in a state of coma. Dr. Weir said he had gone over the brain to find what parts are accessible to surgical interference. We are able to strip up the longitudinal and lateral sinuses to a considerable extent. The *dura mater* may be separated for a considerable distance from the bone. He had been able to raise up the frontal lobes so as to see the anterior clinoid processes, and had been able to feel the *foramen magnum*.

Directing attention now to the other features of the meeting, it may be well to state that the Congress in its incipiency was composed of eleven societies, and at this session has admitted the American Gynecological Society. Two new national societies, the American Pediatric Association and the Association of American Anatomists, were organized during the session and have made application for admission to the Congress. The American Association of Obstetricians and Gynecologists has also made application for admission.

The banquet given at Willard's Hotel was served in sumptuous style. There were 180 covers laid. Two large tables ran almost the entire length of the dining-room, and were connected by a shorter one extending across the room at the end, at which the members of the Committee of Arrangements sat. The center of the table was covered with a bed of flowers on which was the inscription, "American Congress of Physicians and Surgeons, Sept. 17, 1888."

An opportunity was afforded at the banquet to meet Prof. Von Esmarch and other distinguished foreigners. Prof. Esmarch was escorted to the table by Dr. Busey, followed by Dr. Billings and others. The toast, "The Congress," was responded to by

Dr. Billings. "Our Guests" brought out responses from Prof. Esmarch, Sir Spencer Wells and others. "The American Medical Profession" was responded to by Drs. William H. Draper, of New York, Shattuck, of Boston, and D. Hayes Agnew, of Philadelphia.

On Wednesday afternoon, September 19, a reception was given to the members by President and Mrs. Cleveland, and on Thursday evening, after the address of Dr. Billings, the associates and guests, with their ladies, met in a social reunion in the building of the Army Medical Museum and Library.

Von Esmarch has a distinguished appearance, is sixty-five years of age, but his eye is bright and his figure erect. He has a long, flowing white beard.

The final session of the Congress was held in the hall of the National Museum, where Dr. Billings delivered a carefully prepared address, taking for his text "Medical Museums." The second floor of the museum building, including the library and museum halls, were brilliantly lighted and decorated. Flags of all nations were used in great profusion, and the lofty rooms and corridors were glowing with colors.

A great deal of valuable scientific work has been accomplished, and that, it must be confessed, has been the main feature of the sessions of the Congress.

The next meeting will be held in Washington three years from this time. At the next annual meeting of the various societies they will each choose a member to represent them on the Executive Committee, and that committee will select the next President of the Congress. W. W.

*Washington, D. C., September 20, 1888.*

